# Distinct impacts of sleep-disordered breathing on glycemic variability in patients with and without diabetes mellitus

**DOI:** 10.1371/journal.pone.0188689

**Published:** 2017-12-19

**Authors:** Kei Nakata, Takayuki Miki, Masaya Tanno, Hirofumi Ohnishi, Toshiyuki Yano, Atsuko Muranaka, Tatsuya Sato, Hiroto Oshima, Yuki Tatekoshi, Masashi Mizuno, Koki Abe, Tetsuji Miura

**Affiliations:** 1 Department of Cardiovascular, Renal and Metabolic Medicine, Sapporo Medical University School of Medicine, Sapporo, Japan; 2 Department of Public Health, Sapporo Medical University School of Medicine, Sapporo, Japan; 3 Department of Cellular Physiology and Signal Transduction, Sapporo Medical University School of Medicine, Sapporo, Japan; University of Rome Tor Vergata, ITALY

## Abstract

**Background:**

Sleep-disordered breathing (SDB) is highly prevalent in patients with diabetes mellitus (DM) and heart failure (HF) and contributes to poor cardiovascular outcomes. Enlarged glycemic variability (GV) is a risk factor of cardiac events independently of average blood glucose level, but the influence of SDB on GV is uncertain. In this study, we examined whether the impact of SDB on GV is modified by the presence of DM with or without HF.

**Methods and results:**

Two hundred three patients (67.5±14.1 [SD] years old, 132 males) who were admitted to our institute for examination or treatment of DM and/or HF underwent continuous glucose monitoring and polysomnography. Both HbA1c (8.0±2.0 vs. 5.7±0.4%) and mean amplitude of glycemic excursion (MAGE, median: 95.5 vs. 63.5 mg/dl) were significantly higher in a DM group (n = 100) than in a non-DM group (n = 103), but apnea-hypopnea index (AHI: 29.0±22.7 vs. 29.3±21.5) was similar in the two groups. AHI was correlated with log MAGE in the non-DM group but not in the DM group, and multivariate regression analysis revealed that AHI was an independent variable for log MAGE in the non-DM group but not in the DM group. We then divided the non-DM patients into two subgroups according to BNP level (100 pg/ml). AHI was positively correlated with log MAGE (r = 0.74, p<0.001) in the non-DM low-BNP subgroup, but such a correlation was not found in the non-DM high-BNP subgroup. Continuous positive airway pressure (CPAP) reduced MAGE from 75.3 to 53.0 mg/dl in the non-DM group but did not reduce MAGE in the DM group.

**Conclusion:**

Severity of SDB was associated with higher GV, but DM as well as HF diminished the contribution of SDB to GV. Treatment with CPAP was effective for reduction of GV only in patients without DM.

## Introduction

Sleep-disordered breathing (SDB) is a common disorder characterized by repetitive episodes of cessation of breathing leading to intermittent hypoxia, brain arousals, sleep fragmentation, and sympathetic activation. Two main breathing abnormalities in SDB are obstructive sleep apnea (OSA) and central sleep apnea (CSA). Recently, the prevalence of SDB, especially OSA, has been increasing in commensuration with the obesity epidemic [1]. Obesity is significantly associated also with metabolic disorders such as type 2 diabetes mellitus (DM) and impaired glucose tolerance (IGT) [2]. The prevalence of OSA is high in patients with type 2 DM, though it ranges from 18% to 86% in the literature [3–5] possibly due to differences in the definition of OSA and clinical characteristics of study populations [6]. On the other hand, the prevalence of type 2 DM has been reported to be high in patients with OSA [7,8], suggesting that there is bidirectional association between OSA and type 2 DM. However, DM is not the only comorbidity of SDB. Cardiovascular diseases, including chronic heart failure (HF), are known to be associated with increased SDB [9–11], and SDB is potentially a risk factor for mortality in HF patients [12,13]. HF is known to reduce insulin sensitivity regardless of the presence or absence of SDB, and DM is an established risk factor of HF [14]. Thus, it is reasonable to postulate mutual relationships between SDB, glucose intolerance and HF, but the relationships have not been characterized.

In addition to levels of plasma glucose and HbA1c, glycemic variability (GV) has recently received attention because frequent and/or large glucose fluctuations have been suggested to contribute to diabetes-related complications [15–18]. However, it is difficult to accurately estimate GV from HbA1c and plasma glucose levels, particularly in patients with poor metabolic control [19,20]. Continuous glucose monitoring (CGM) is a minimally invasive method that has been approved for ambulant glucose monitoring and is a useful tool to assess GV in patients with DM. Differences in GV before and after treatment of OSA with continuous positive airway pressure (CPAP) in DM patients have been examined in several studies [21–25]. However, to our knowledge, there has been no study in which the impact of SDB on GV in non-DM subjects was examined in detail, though it is reasonable to assume that neurohumoral responses to SDB have an influence on GV. Thus, in the present study, we examined whether severity of SDB is associated with GV and whether the association, if any, is modified by the presence of DM and/or HF.

## Materials and methods

This study was conducted in strict adherence with the principles of the Declaration of Helsinki and was approved by the Clinical Investigation Ethics Committee of Sapporo Medical University Hospital. Written, informed consent was obtained from all participants.

### Study subjects

We prospectively enrolled patients who were admitted to our institute from July 2013 to October 2016. Inclusion criteria were admission for examination or management of DM and/or HF and consent for both polysomnography and CGM. Exclusion criteria were type 1 DM and other specific types of DM, presence of sinusitis, hypertrophy of the tongue or tonsils, endocrine disorders and malignancy. Two hundred thirty-three patients met the inclusion criteria, and 19 and 11 patients were excluded by the exclusion criteria and due to missing data of CGM or polysomnography, respectively. Thus, 203 patients contributed to the present analyses. None of these patients had any previous experience of CPAP. The definition of DM in the present study included being on oral hypoglycemic agents or insulin or HbA1c≥6.5%. Patients who do not fall under the criteria for DM were designated as non-DM subjects. After completion of clinical examinations or standard therapy for DM and/or HF, patients underwent CGM for 3 days and overnight polysomnography. The patients took 1400 kcal-1600 kcal meals depending on their standard body weight during hospital admission.

### Polysomnography

Nocturnal polysomnography was performed with multichannel monitoring including an electroencephalogram, electro-oculogram, submental electromyogram, electrocardiogram, and measurement of thoraco-abdominal motion, nasal pressure, and peripheral capillary oxygen saturation (SpO_2_) by pulse oximetry. Continuous recordings were obtained with a computerized diagnostic system (AlicePDx®: Philips Respironics, Tokyo, Japan). The sleep record was manually analyzed by physicians. Central apnea was defined as an absence of oronasal airflow during sleep for ≥10 s associated with absent respiratory effort. Obstructive apnea was defined as an absence of oronasal airflow for ≥10 s in the presence of thoraco-abdominal effort. The apnea-hypopnea index (AHI) was calculated as the mean number of occurrence of apnea and hypopnea per hour of sleep. SDB severity categories were defined according to commonly used clinical cutoffs as follows: no SDB (AHI<5); mild SDB (5≤AHI<15); moderate SDB (15≤AHI<30) and severe SDB (AHI≥30).

### Glucose monitoring

Interstitial glucose levels were monitored using the CGM System (iPro2®, Medtronic, Northridge, CA), which records glucose level every 5 min for up to 72 h. Sensor calibration was accomplished by self-monitored blood glucose values measured by OneTouch UltraVue (Johnson & Johnson, Tokyo, Japan). The mean blood glucose level, standard deviation (SD), mean amplitude of glycemic excursion (MAGE), percentage of time at blood glucose <70.2 mg/dl, and percentage of time at blood glucose ≥180 mg/dl were measured from the data recorded through CGM.

### Intervention

If patients were diagnosed with moderate or severe SDB, we suggested that the patients receive CPAP treatment as a part of standard care for SDB. Patients who agreed to undergo CPAP therapy were provided with an auto-titrating device (REMstar Auto System One, dream station Auto: Philips Respironics) by a qualified technician. The patients received usual care at outpatient clinics after discharge from our institute. The patients were re-admitted to our institute several months after commencement of CPAP treatment for CGM under the same meal condition as that during the previous hospitalization without CPAP.

### Statistical analysis

Numeric variables are expressed as means±SD. Variables with non-normal distributions are shown as medians and interquartile range (IQR), and we used log transformation for these variables in linear regression analyses. Differences between two groups were tested by Student’s *t*-test or the Mann-Whitney U test. The Kruskal-Wallis test followed by the Mann-Whitney U test was used for comparison among three groups. Relationships between parameters were examined by the use of simple linear regression analyses. Changes in log MAGE, severity of SDB and clinical and glycemic variables during follow-up periods in the CPAP-treated group were compared by the paired *t*-test or Wilcoxon’s rank sum test. To determine the independent associations of parameters with log MAGE, we performed univariate and multivariate linear regression analyses with stepwise model selection. Statistical analyses were carried out using JMP (version11 SAS Institute, Cary, NC, USA). All statistical tests were two-tailed and differences were considered to be statistically significant if p was less than 0.05.

## Results

### Baseline characteristics

The mean age of the patients (n = 203) was 67.5±14.1 years, and 65.0% of them were male. The patients were divided into a DM group and a non-DM group. Baseline characteristics of the patients are shown in Table 1. Body mass index (BMI) was larger and systolic blood pressure (BP) was higher in the DM group than in the non-DM group. However, BP was relatively well-controlled by medications in most of the DM patients (123.0±18.8/70.0±12.0 mmHg). Fasting plasma glucose (FPG: 138.3±53.7 vs. 91.2±13.6 mg/dl), HbA1c (8.0±2.0% vs. 5.7±0.4%) and homeostasis model assessment ratio (HOMA-R: 2.69±2.71 vs. 1.48±1.17) were significantly higher in the DM group than in the non-DM group. As for DM complications, 36.0%, 58.0%, and 53.0% of the patients had retinopathy, neuropathy and nephropathy, respectively. Triglyceride level was higher and high-density lipoprotein cholesterol level was slightly lower in the DM patients than in the non-DM patients. Low-density lipoprotein cholesterol levels were comparable in the two groups, most likely as a result of statin therapy in half of the DM patients. Because non-DM patients in this study were the subjects who did not meet the diagnostic criteria of DM, they are not necessarily “healthy normal” subjects. The proportion of patients with HF was higher in the non-DM group than the DM group. Thus, left ventricular ejection fraction (LVEF) was slightly lower and brain natriuretic peptide (BNP) level was slightly higher in the non-DM group, though the differences were not statistically significant. Of 103 non-DM patients, 7 had high fasting plasma glucose (≥110 mg/dl), but they did not meet the diagnostic criteria for DM in the Japan Diabetes Society.

**Table 1:** Baseline clinical characteristics.

Variables	ALL	DM	Non-DM	P value
	(n = 203)	(n = 100)	(n = 103)	
Age, years	67.5±14.1	66.0±13.9	68.8±14.1	0.14
Male (%)	132 (65.0)	67 (67.0)	65 (63.1)	0.56
BMI, kg/m^2^	25.1±5.4	26.3±5.6	23.9±4.8	<0.01
Systolic BP, mmHg	119.1±18.6	123.0±18.8	115.2±17.8	<0.01
Diastolic BP, mmHg	68.5±13.1	70.0±12.0	67.0±14.1	0.10
Hypertension (%)	146 (71.9)	65 (65.0)	81 (78.6)	<0.01
Dyslipidemia (%)	104 (51.2)	65 (65.0)	39 (37.8)	<0.01
HF (%)	111 (54.7)	45 (45.0)	66 (64.1)	<0.01
Ischemic HF (%)	35 (31.5)	18 (40.0)	17 (25.8)	0.78
Nonischemic HF (%)	76 (68.5)	27 (60.0)	49 (77.8)	<0.01
NYHA class				
I	60	33	27	0.43
II	27	14	13	0.90
III	42	11	31	0.06
IV	12	5	7	0.36
LVEF, %	52.6±16.5	61.7±10.0	50.8±16.1	0.11
Retinopathy (%)		36 (36.0)	N/A	
Neuropathy (%)		58 (58.0)	N/A	
Nephropathy (%)		53 (53.0)	N/A	
Medications (%)				
CCB (%)	78 (38.4)	44 (44.0)	34 (33.0)	0.12
ACE-I/ARB (%)	117 (57.6)	64 (64.0)	53 (51.5)	0.18
β blocker (%)	114 (56.2)	48 (48.0)	66 (64.1)	<0.01
Diuretics (%)	110 (54.2)	50 (50.0)	60 (58.2)	0.24
Statin (%)	84 (41.4)	50 (50.0)	34 (33.0)	0.01
Sulphonylurea (%)		15 (15.0)	N/A	
α-glucosidase inhibitor (%)		19 (19.0)	N/A	
Biguanide (%)		21 (21.0)	N/A	
DPP-4 inhibitor (%)		62 (62.0)	N/A	
Insulin (%)		35 (35.0)	N/A	
Other antidiabetic drugs (%)		17 (17.0)	N/A	
Laboratory variables				
FPG, mg/dl	114.4±45.4	138.3±53.7	91.2±13.6	<0.01
HbA1c, %	6.8±1.8	8.0±2.0	5.7±0.4	<0.01
HOMA-R	2.08±2.16	2.69±2.71	1.48±1.17	<0.01
Triglyceride, mg/dl	113 (77–170)	134 (87–186)	103 (73–145)	<0.01
HDL-C, mg/dl	48.9±17.1	46.9±15.2	50.8±18.6	0.10
LDL-C, mg/dl	99.4±40.6	102.3±47.9	96.5±32.0	0.31
Creatinine, mg/dl	1.00 (0.74–1.30)	1.05 (0.71–1.48)	0.94 (0.74–1.22)	0.23
Hb, g/dl	12.8±2.4	13.1±2.3	12.6±2.4	0.20
BNP, pg/ml	132.7 (28.6–430.0)	62.1 (17.0–415.9)	186.6 (43.7–482.8)	0.28

Data are presented as means±SD, No. (%) or median (IQR)

BMI = body mass index, BP = blood pressure, HF = heart failure, NYHA = New York Heart Association

LVEF = left ventricular ejection fraction, N/A = not applicable, CCB = calcium channel blocker

ACE-I = angiotensin-converting enzyme inhibitor, ARB = angiotensin II receptor blocker

DPP-4 = dipeptidyl peptidase-4, FPG = fasting plasma glucose, HbA1c = glycated hemoglobin

HOMA-R = homeostasis model assessment ratio, HDL-C = high-density lipoprotein cholesterol

LDL-C = low-density lipoprotein cholesterol, Hb = hemoglobin, BNP = brain natriuretic peptide

### Sleep and respiratory characteristics

Overnight polysomnographic data are shown in Table 2. Although more than half of the patients had moderate-severe SDB, Epworth Sleepiness Scale scores were relatively low. Total sleep time, time of waking after sleep onset, and sleep efficiency were similar in the DM group and non-DM group. Average AHI was high in patients in the present study, but the indexes were comparable in the two groups (29.0±22.7 vs. 29.3±21.5). Not only AHI but also 3% oxygen desaturation index (ODI), time at SpO_2_<90% and lowest SpO_2_ were similar in the two groups.

**Table 2:** Sleep and respiratory characteristics.

Variables	ALL	DM	Non-DM	P value
	(n = 203)	(n = 100)	(n = 103)	
Epworth Sleepiness Scale score	6.0±3.4	5.7±3.3	6.2±3.5	0.39
Total sleep time, min	337.9±120.4	329.4±116.9	346.2±123.7	0.33
WASO, min	123.3±90.6	127.1±95.9	119.4±85.2	0.55
Sleep efficiency, %	63.4±23.8	64.0±26.7	62.8±20.6	0.72
Arousal Index, no./h	24.9±18.1	24.8±19.0	25.0±17.2	0.92
AHI, no./h	29.3±22.0	29.0±22.7	29.3±21.5	0.92
AHI<5	12 (5.9)	9 (9.0)	3 (2.9)	0.55
AHI≥5, <15	53 (26.1)	26 (26.0)	27 (26.2)	0.77
AHI≥15, <30	59 (29.1)	29 (29.0)	30 (29.1)	0.77
AHI≥30	79 (38.9)	36 (36.0)	43 (41.8)	0.24
Apnea, no./h	14.3±17.7	12.9±16.3	15.3±18.7	0.32
Central apnea, no./h	3.3±8.3	2.6±7.9	3.6±8.2	0.33
Obstructive apnea, no./h	8.2±12.4	7.8±11.0	8.5±13.6	0.68
3%ODI, no./h	25.3±21.8	26.0±216	24.3±22.1	0.58
Time at SpO_2_<90%, min	29.7±70.5	29.8±54.6	29.5±83.9	0.97
Lowest SpO_2_, %	85 (79–89)	83 (78–88)	86 (78–88)	0.22

Data are presented as means±SD, No. (%) or median (IQR)

WASO = waking after sleep onset, AHI = apnea-hypopnea index

ODI = oxygen desaturation index, SpO_2_ = peripheral capillary oxygen saturation

### GV measured by CGM

As shown in Table 3, mean blood glucose level was significantly higher in the DM group than in the non-DM group (148.8±33.2 vs. 117.6±12.8 mg/dl). Parameters for GV including MAGE, SD, and percentage coefficient of variation (%CV) were significantly higher in the DM group than in the non-DM group. Few hypoglycemic episodes, i.e., interstitial glucose level lower than 70.2 mg/dl, were observed in either of the groups.

**Table 3:** Glycemic variables.

Variables	ALL	DM	Non-DM	P value
	(n = 203)	(n = 100)	(n = 103)	
Mean blood glucose, mg/dl	133.0±30.0	148.8±33.2	117.6±12.8	<0.01
MAGE, mg/dl	76.6 (55.0–104.5)	95.5 (80.0–117.4)	63.5 (46.6–76.5)	<0.01
SD, mg/dl	27.1±11.8	32.8±12.0	21.5±8.5	<0.01
%CV, mg/dl	20.1±6.9	22.0±6.4	18.3±6.9	<0.01
M100	7.3±12.6	12.3±16.3	2.5±2.7	<0.01
J-INDEX	27.1±15.2	34.7±18.0	19.7±5.2	<0.01
>180 mg/dl, % of total	11.5±19.2	21.0±23.2	2.4±5.4	<0.01
<70.2 mg/dl, % of total	1.8±3.8	1.2±2.9	2.4±4.5	<0.01
Mean blood glucose-nht, mg/dl	120.5±29.6	128.8±32.5	103.6±13.3	<0.01
MAGE-nht, mg/dl	40.1 (26.1–61.9)	49.6 (33.8–80.8)	31.0 (20.0–49.4)	<0.01
SD-nht, mg/dl	17.9±10.9	20.5±11.4	12.1±6.8	<0.01
>180 mg/dl-nht, % of total	6.7±17.6	10.3±21.5	0.0±0.3	<0.01
<70.2 mg/dl-nht, % of total	3.8±8.8	3.2±8.0	5.3±10.4	0.11

Data are presented as means±SD or median (IQR)

MAGE = mean amplitude of glycemic excursions, SD = standard deviation

%CV = percentage coefficient of variation, M100 = weighted average of glucose values

nht = during night time (0:00–6:00 am)

### Relationship between SDB and GV

#### DM patients vs. non-DM patients

**Fig 1:**
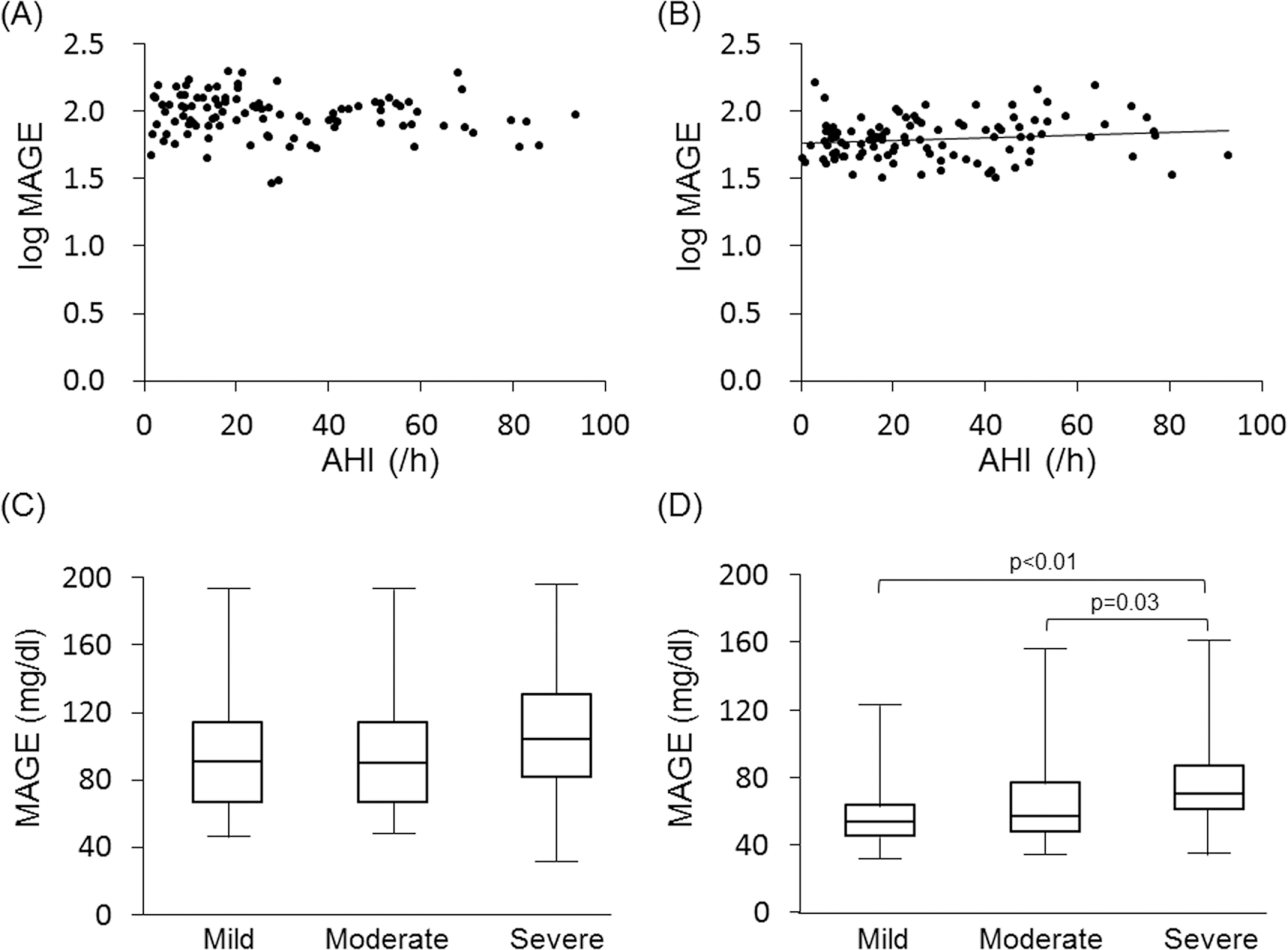
Relationship between severity of SDB and glucose variability: DM group vs. non-DM group. Scatterplots of log MAGE against AHI in the DM group (A) and in the non-DM group (B). There was no correlation between AHI and log MAGE in the DM group, but there was a weak correlation in the non-DM group (y = 0.001x+1.8, r = 0.14, p<0.01). Comparisons of MAGE between subgroups with different severities of SDB in the DM group (C) and in the non-DM group (D). SDB was classified by its severity into mild SDB (5≤AHI<15), moderate SDB (15≤AHI<30) and severe SDB (AHI≥30). Level of MAGE in the severe SDB subgroup was significantly higher than that in the mild SDB subgroup in the non-DM group but not in the DM group. The ends of the closed box show the upper and lower quartiles, and the upper and lower whiskers represent data outside the middle 50%.

Indices of SDB severity (i.e., AHI, 3%ODI and lowest SpO_2_) were not correlated with parameters of GV (MAGE, SD or %CV) in analysis of data for all patients. However, a weak correlation between AHI and log MAGE was found in the non-DM group (r = 0.14, p<0.01) but not in the DM group (Fig 1A and 1B). When the patients were divided into three subgroups by severity of SDB, MAGE in the severe SDB subgroup (i.e., AHI≥30) was significantly higher than those in the mild SDB subgroup (5≤AHI<15) and moderate SDB subgroup (15≤AHI<30) in the non-DM patients, but such a difference was not observed in the DM patients (Fig 1C and 1D). In multivariate regression analysis for log MAGE, age and insulin use were selected as independent variables in the DM group, whereas age, HOMA-R and AHI were selected as independent variables in the non-DM group (Tables 4 and 5).

**Table 4:** Univariate and multivariate regression analysis for log MAGE in the DM group.

	Univariate analysis			Multivariate analysis	
Variables	β	SE	P value	β	P value
Age	0.003	0.001	<0.01	0.003	<0.01
Male	-0.015	0.003	0.63	-0.010	0.76
BMI	-0.005	0.003	0.08	-	
Systolic BP	0.001	0.001	0.16	-	
HbA1c	-0.001	0.008	0.88	-	
FPG	0.000	0.000	0.42	-	
HOMA-R	-0.007	0.006	0.25	-	
log Triglyceride	-0.055	0.060	0.40	-	
HDL-C	0.001	0.001	0.39	-	
LDL-C	0.000	0.000	0.35	-	
log Creatinine	0.110	0.065	0.09	-	
Hb	-0.008	0.007	0.24	-	
log BNP	0.032	0.020	0.10	-	
LVEF	0.000	0.001	0.64	-	
Epworth Sleepiness Scale score	0.001	0.005	0.76	-	
AHI	0.001	0.001	0.26	0.000	0.59
3% ODI	0.001	0.001	0.42	-	
Time at SpO_2_<90%	0.000	0.003	0.62	-	
log lowest SpO_2_	0.096	0.373	0.80	-	
β-blocker	0.012	0.030	0.71	-	
Sulphonylurea	0.007	0.045	0.89	-	
α-glucosidase inhibitor	-0.003	0.040	0.93	-	
Biguanide	-0.088	0.038	0.02	-0.040	0.28
DPP-4 inhibitor	0.039	0.033	0.24	-	
Insulin	0.110	0.030	<0.01	0.100	<0.01
Other antidiabetic drugs	0.047	0.080	0.57	-	

BMI = body mass index, BP = blood pressure, FPG = fasting plasma glucose, HbA1c = glycated hemoglobin

HOMA-R = homeostasis model assessment ratio, HDL-C = high-density lipoprotein cholesterol

LDL-C = low-density lipoprotein cholesterol, Hb = hemoglobin, BNP = brain natriuretic peptide

LVEF = left ventricular ejection fraction, AHI = apnea-hypopnea index, ODI = oxygen desaturation index

SpO_2_ = peripheral capillary oxygen saturation, DPP-4 = dipeptidyl peptidase-4

**Table 5:** Univariate and multivariate regression analysis for log MAGE in the non-DM group.

	Univariate analysis			Multivariate analysis	
Variables	β	SE	P value	β	P value
Age	0.003	0.001	<0.01	0.003	0.01
Male	-0.023	0.031	0.47	-0.046	0.11
BMI	0.002	0.003	0.48	-	
Systolic BP	0.000	0.000	0.78	-	
HbA1c	0.108	0.043	0.02	0.079	0.06
FPG	0.002	0.001	0.19	-	
HOMA-R	0.036	0.013	<0.01	0.037	<0.01
log Triglyceride	0.039	0.065	0.55	-	
HDL-C	0.000	0.000	0.44	-	
LDL-C	0.000	0.000	0.34	-	
log Creatinine	-0.005	0.073	0.94	-	
Hb	-0.007	0.006	0.29	-	
log BNP	0.011	0.020	0.59	-	
LVEF	0.001	0.001	0.30	-	
Epworth Sleepiness Scale score	0.004	0.004	0.38	-	
AHI	0.003	0.001	<0.01	0.002	<0.01
3% ODI	0.002	0.001	<0.01	-	
Time at SpO_2_<90%	0.000	0.000	0.02	-	
log lowest SpO_2_	-0.600	0.300	0.05		
β-blocker	0.025	0.027	0.37	-	

BMI = body mass index, BP = blood pressure, FPG = fasting plasma glucose, HbA1c = glycated hemoglobin

HOMA-R = homeostasis model assessment ratio, HDL-C = high-density lipoprotein cholesterol

LDL-C = low-density lipoprotein cholesterol, Hb = hemoglobin, BNP = brain natriuretic peptide

LVEF = left ventricular ejection fraction, AHI = apnea-hypopnea index, ODI = oxygen desaturation index

SpO_2_ = peripheral capillary oxygen saturation

#### Patients with low BNP level vs. patients with high BNP level

**Fig 2:**
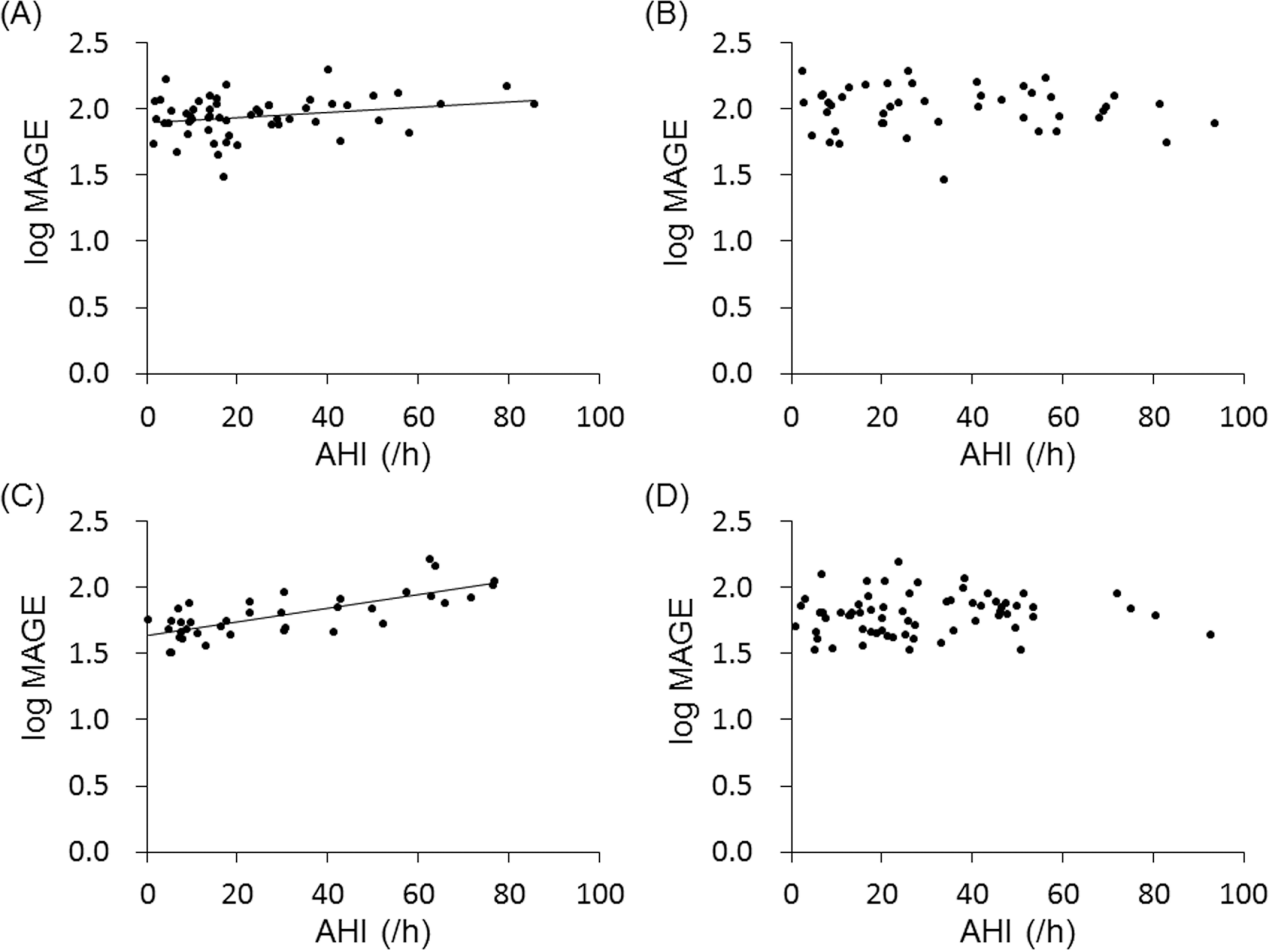
Relationship between severity of SDB and glucose variability in subgroups with different BNP levels. Scatterplots of log MAGE against AHI in the DM low-BNP subgroup (A), DM high-BNP subgroup (B), non-DM low-BNP subgroup (C) and non-DM high-BNP subgroup (D). Significant correlations were observed in the DM low-BNP subgroup (y = 0.002x+1.9, r = 0.26, p<0.05) and non-DM low-BNP subgroup (y = 0.005x+1.6, r = 0.74, p<0.01).

**Fig 3:**
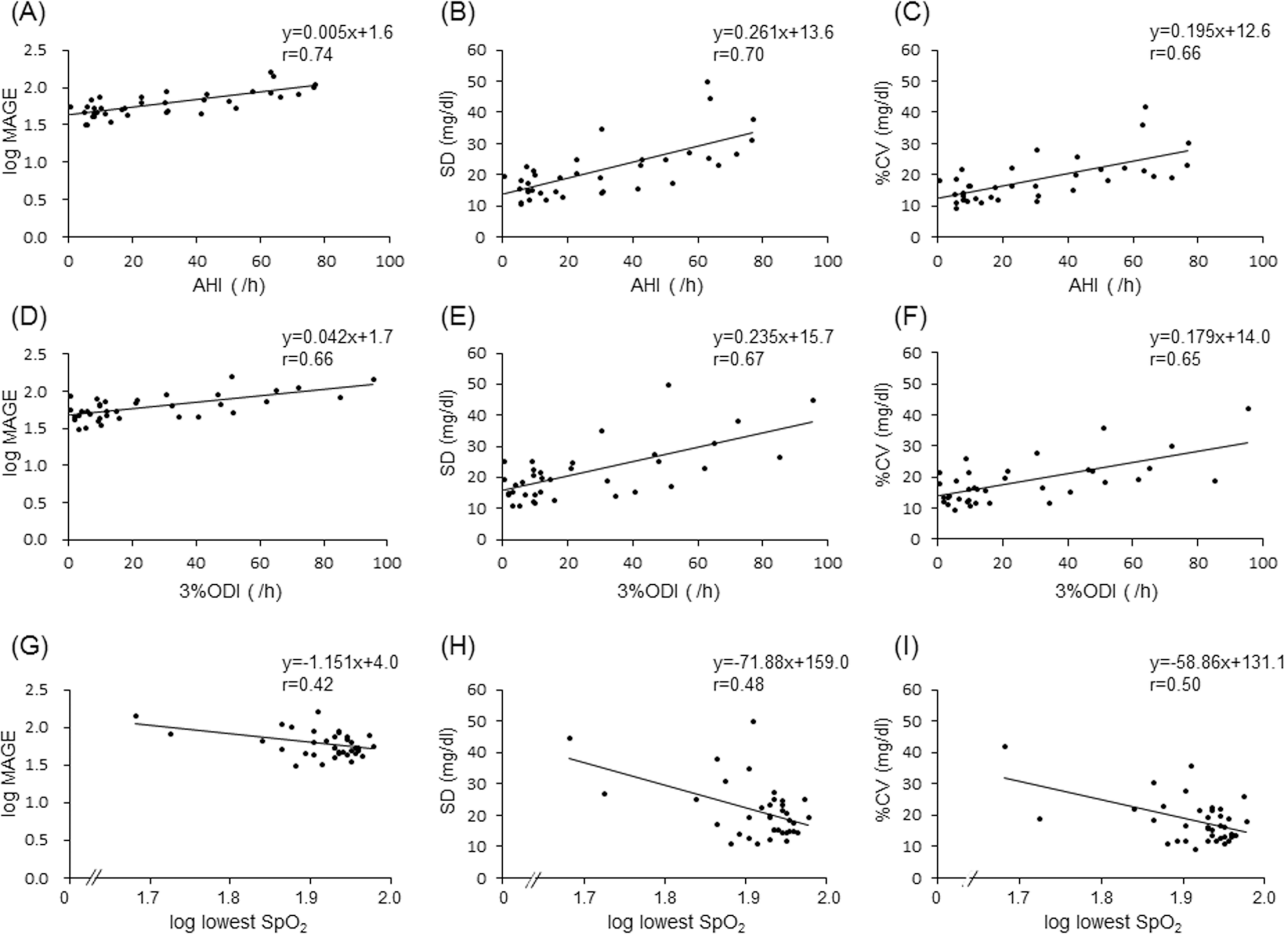
Relationships between severity of SDB and indices of glucose variability. Log MAGE (A), SD (B) and %CV (C) in each patient were plotted against AHI (A, B, C), 3%ODI (D, E, F) and log lowest SpO_2_ (G, H, I) in the non-DM low-BNP group. Of these relationships, the correlation between AHI and log MAGE was strongest.

To examine the influence of HF on the relationship between SDB and GV, we divided the patients according to BNP level into four subgroups: DM low-BNP, DM high-BNP, non-DM low-BNP, and non-DM high-BNP subgroups. We used 100 pg/ml of BNP as a cutoff point to divide patients into low- and high-BNP subgroups, since 100 pg/ml of BNP is a useful level not only for diagnosis of HF but also for prediction of cardiovascular events [26,27]. In the DM group, a weak association was found between AHI and log MAGE among patients with BNP< 100 pg/ml (DM low-BNP subgroup: r = 0.26, p<0.05, Fig 2A) but not among patients with BNP≥100 pg/ml (Fig 2B). In the non-DM group, HbA1c (5.6±0.3 vs. 5.8±0.4 mg/dl), MAGE (median, 55.1 vs. 63.8 mg/dl) and AHI (29.7±24.2 vs. 29.1±20.1) were similar in the low-BNP and high-BNP subgroups. AHI was strongly correlated with log MAGE (r = 0.74, p<0.01) in the non-DM low-BNP subgroup, but such a correlation was not found in the non-DM high-BNP subgroup (Fig 2C and 2D). These results suggest that severity of SDB is closely associated with higher GV, but this relationship was diminished by the presence of DM or HF. Similar to AHI, other SDB indices, i.e., 3%ODI and lowest SpO_2_ (log lowest SpO_2_), were correlated with MAGE (log MAGE), SD and %CV in the non-DM low-BNP group, but a correlation between AHI and MAGE was strongest in the present study (Fig 3).

### CPAP therapy study

Of the 203 enrolled patients, 59 patients received CPAP therapy and 28 of them (15 DM patients and 13 non-DM patients) agreed to re-admission to our institute for evaluation of GV by CGM under the same meal condition as that during their first admission. Compared with data for total study subjects (Table 1), AHI was larger in patients enrolled in the CPAP therapy study, especially in those without DM (AHI: 37.3±18.0 in the DM group and 49.1±16.1 in the non-DM group, Table 6). MAGE before CPAP therapy in non-DM patients was higher than the average value for all non-DM patients, probably reflecting the severity of SDB (median MAGE, 75.3 vs. 63.5 mg/dl). The durations of CPAP therapy were 104.4±114.1 and 87.0±109.2 days and the CPAP usage times were 5.3±2.3 and 6.5±1.8 h/night in the DM group and non-DM group, respectively. HbA1c and mean blood glucose were not changed after CPAP treatment in either group. Three indices of GV, MAGE, SD and %CV, were significantly improved by CPAP in the non-DM group. A favorable effect of CPAP on GV was also observed in the DM group, but it did not reach statistical significance. Systolic BP was decreased, but BMI, lipids and BNP were not changed after CPAP treatment.

**Table 6:** Clinical parameters before and after CPAP.

Variables	DM (n = 15)			Non-DM (n = 13)		
	Before CPAP	After CPAP	P value	Before CPAP	After CPAP	P value
Age, years	74.2±7.7	-	-	68.1±10.2	-	-
Male	10 (66.7)	10 (66.7)	-	10 (76.9)	10 (76.9)	-
BMI, kg/m^2^	25.5±4.3	25.5±4.3	0.86	27.3±3.6	27.0±3.9	0.82
Systolic BP, mmHg	131.1±19.1	118.8±15.0	0.02	125.1±13.3	113.1±10.4	0.03
Diastolic BP, mmHg	67.1±13.4	64.1±8.5	0.26	66.9±17.2	67.6±9.3	0.86
Laboratory variables						
HbA1c, %	7.1±1.7	7.0±1.4	0.73	5.7±0.3	5.9±0.6	0.05
HOMA-R	2.35±1.39	2.32±1.75	0.95	2.62±2.43	2.31±2.36	0.07
Triglyceride, mg/dl	106 (71–151)	108 (85–160)	0.33	101 (65–166)	95 (83–171)	0.72
HDL-C, mg/dl	47.7±11.9	45.9±8.7	0.50	46.7±11.4	48.5±9.1	0.32
LDL-C, mg/dl	87.7±38.5	96.0±39.9	0.49	94.6±30.0	104.6±33.4	0.24
BNP, pg/ml	57.4 (19.9–194.7)	81.9 (17.1–129.0)	0.23	72.6 (23.7–197.6)	56.6 (9.0–72.9)	0.41
Sleep variables						
AHI, no./h	37.3±18.0	7.2±6.5	<0.01	49.1±16.1	7.0±5.5	<0.01
CPAP usage time, hr/night	-	5.3±2.3	-	-	6.5±1.8	-
CPAP treatment duration, day	-	104.4±114.1	-	-	87.0±109.2	-
Glycemic variables						
Mean blood glucose, mg/dl	145.7±30.7	148.9±39.6	0.74	117.2±12.6	117.3±10.7	0.51
MAGE, mg/dl	78.6 (74.9–125.0)	71.8 (49.2–115.6)	0.07	75.3 (58.2–101.3)	52.9 (43.1–69.2)	0.01
SD, mg/dl	32.4±12.7	27.4±13.4	0.12	25.8±11.7	18.1±5.2	<0.01
%CV, mg/dl	22.4±8.2	18.5±8.0	0.09	22.0±9.3	15.4±3.8	<0.01

Data are presented as means±SD, No. (%) or median (IQR)

BMI = body mass index, BP = blood pressure, HbA1c = glycated hemoglobin, HOMA-R = homeostasis model assessment ratio

HDL-C = high-density lipoprotein cholesterol, LDL-C = low-density lipoprotein cholesterol, BNP = brain natriuretic peptide

AHI = apnea-hypopnea index, CPAP = continuous positive airway pressure, MAGE = mean amplitude of glycemic excursions

SD = standard deviation, %CV = percentage coefficient of variation

## Discussion

Although the impact of SDB on average level of blood glucose has been studied in patients with type 2 DM and also in patients with normal glucose tolerance [3,28,29], there has been no study that focused on change in GV caused by SDB. The present study showed for the first time that SDB had a significant impact on GV and that the impact was different depending on the presence or absence of DM. There was a weak but significant correlation between AHI and log MAGE in the non-DM group but not in the DM group (Fig 1). In the non-DM group, SDB markers, including AHI, were associated with log MAGE, as were HbA1c and HOMA-R, by univariate analysis, and AHI was selected as an independent variable for log MAGE by multivariate analysis (Table 5). In contrast to the findings in non-DM patients, age and use of biguanide or insulin were associated with log MAGE by univariate analysis in patients with DM, and insulin use was selected as an independent variable for log MAGE (Table 4). None of the SDB markers was associated with log MAGE in the DM group (Table 4). We also did not find significant relationships between SDB markers and log MAGE when the DM patients were divided by insulin usage or duration of DM in the present study (S1 Fig). These results suggest that SDB increases GV depending on its severity, though the contribution of SDB to GV is diminished by the presence of DM. The influences of poor glycemic control itself and/or use of antidiabetic agents, such as insulin [30], may overwhelm the effect of SDB on GV in DM patients.

In the present study, median MAGE in the non-DM patients seems to be higher than those in earlier reports [31–33]. Zhou et al. and Gude et al. have reported that median MAGE for non-DM subjects was 31 mg/dl and 26 mg/dl, respectively. However, the subjects in those reports were relatively young and healthy people who were not only without diabetes but also without other metabolic disease and coronary artery disease. MAGE may be influenced by concomitant diseases, since MAGE for non-DM patients with coronary artery disease was reported to be high (60.1±32.9 mg/dl) [34]. Since non-DM patients in the present study were older and had high rates of history of metabolic and cardiac diseases (Table 1), MAGE in this group was relatively higher than that in the healthy control subjects in the previous reports [31–33].

As in the case of SDB, HF is closely associated with activation of sympathetic nerves, increased levels of proinflammatory mediators and oxidative stress, leading to insulin resistance and endothelial dysfunction [14,35–37]. Therefore, it is plausible that the presence of HF increases GV and modifies the association between SDB and GV. However, we did not find a significant correlation between BNP or LVEF and log MAGE in either the DM group and non-DM group (Tables 4 and 5). Similarly, Dungan et al. [38] reported that HF was not associated with increased GV, even though plasma catecholamine was higher in patients with HF than in those without HF. However, the findings do not exclude the possibility that the impact of HF on GV was masked by other factors influencing GV. In fact, as shown in Fig 2, a significant correlation of log MAGE with AHI was observed in patients with BNP<100 pg/ml, and the correlation coefficient was larger in a subgroup without DM than in a subgroup with DM. In multivariate regression analysis for log MAGE, age and AHI were selected as independent variables in the non-DM low-BNP subgroup, though none of the SDB markers were selected in the non-DM high-BNP subgroup (S1 Table and S2 Table). AHI was not selected as an independent variable for log MAGE in patients with DM regardless of the BNP level (S3 Table and S4 Table). Taken together, the results suggest that an increase in GV by SDB is masked by left ventricular dysfunction reflected by elevation of BNP.

CPAP is the gold standard therapy for SDB, especially for OSA. Although conflicting results have been shown by earlier studies, several meta-analyses have characterized the effects of CPAP on glycemic control in OSA patients [39–41]. CPAP was shown to improve insulin resistance without significant change in FPG and BMI in non-DM patients with OSA (39). Such a benefit of CPAP for insulin sensitivity was shown also in DM patients with OSA, but an improvement of HbA1c by CPAP appears to be achieved only when patients adequately adhered to CPAP treatment for a long period [40]. However, impact of CPAP on GV was not addressed in the meta-analyses [39–41].

To date, the effect of CPAP on GV determined by CGM in type 2 DM patients has been examined in five studies [22–25,42]. A significant reduction in GV that was assessed by MAGE or SD after CPAP treatment was reported by Pallayova et al. [23] and Guo et al. [24] but not by the other investigators [22,25,42]. Pallayova et al. reported that SD and %CV were decreased rapidly with the initiation of CPAP therapy (i.e., during a CPAP titration night) [22]. In a study by Guo et al. [30], 30 days of CPAP reduced MAGE and SD by 29% and 27%, respectively, in addition to reduction of HbA1c from 8.70% to 6.95% [24]. In the present study, adherence to CPAP was adequate (> 4 h/night), and the duration of CPAP was about 3 months. Examinations of CGM were repeated under the condition of the same meals in the same institute. GV markers, including MAGE, SD and %CV, tended to be reduced in DM patients, but the changes were not statistically significant (Table 6). There is no clear explanation for the discrepant results, but differences in meals during CGM (i.e., meals at home vs. meals during hospital admission), severity of SDB and proportion of study subjects with HF might have been involved. Additionally, a small contribution of SDB to GV in DM patients (Fig 2A and 2B) may be an explanation for the inconsistent impact of CPAP on GV in the earlier studies and the present study. We found that CPAP significantly improved GV markers in non-DM SDB patients (Table 6). To our knowledge, there has been no study in which the effect of CPAP on GV assessed by CGM in non-DM subjects was examined. The mechanism of the improvement in GV after CPAP treatment remains unclear, but some speculations are possible. HOMA-R was unchanged after CPAP, but it predominantly reflects hepatic insulin sensitivity and it is not as sensitive to change in whole body insulin resistance as is Matsuda-DeFronzo index or M value obtained by a euglycemic glucose clamp [43]. Thus, improvement in insulin sensitivity of the skeletal muscle and change in the variability of insulin or glucagon secretion or glucose demand are possible mechanisms underlying the improvement in GV by CPAP in non-DM SDB patients. However, there is no direct evidence for any of the possibilities, and this issue needs to be further investigated.

The results of the present study have important clinical implications. Prevention and treatment of cardiac dysfunction is important to maximize the benefit of CPAP on GV control. As shown in Fig 2, GV seems to be insensitive to improvement of AHI by CPAP when BNP is elevated above 100 pg/ml. In fact, asymptomatic ventricular dysfunction caused by “diabetic cardiomyopathy” is not rare in DM patients [44] and should be considered in treatment of diabetic SDB patients with CPAP.

There are limitations in this study. First, patients enrolled in the present study were those who were admitted to our institute for examination or management of DM and/or HF but not for diagnosis for SDB. In addition, only 28 (14%) of the total of 203 patients agreed to re-admission for CGM study after induction of CPAP therapy. Therefore, selection bias of patients might have affected the results of the present study. Second, since a history of SDB and thus the temporal relationship between SDB and DM or HF were unclear in the study subjects, it was difficult to clarify the causal relationships between SDB, DM and HF. Third, the protocol of treatment for DM or HF was not pre-specified, though treatment of both diseases was in accordance with treatment guidelines of the Japanese Diabetic Society and Japanese Circulation Society. Thus, variations in the treatment regimen might have modified the relationships between indices of SDB and GV. Finally, we did not measure indices of sympathetic nervous activation, inflammatory cytokines or oxidative stress at the time of polysomnography and CGM. Thus, the results of the present study provide few mechanistic insights into modification of the SDB-GV association by DM and HF.

## Conclusions

Severe SDB was associated with higher GV, but the presence of DM or HF diminished the contribution of SDB to GV. CPAP therapy significantly reduced severity of SDB, but it improved GV only in patients without DM. Whether stabilization of GV by treatment of SDB has a clinical benefit for prevention of cardiac events remains to be further investigated.

## Supporting information

S1 Fig Relationship between severity of SDB and glucose variability in DM group: Effects of insulin usage and duration of DM. Scatterplots of log MAGE against AHI in the DM patients treated with insulin (A) and those without insulin (B). There was no correlation between AHI and log MAGE in the DM group regardless of insulin usage. Scatterplots of log MAGE against AHI in patients with short-duration DM (<7 years [median]: C) and those with long-duration DM (> 7 years [median]: D). Log MAGE was not correlated with AHI even in patients with short-duration DM.
(TIF)

S1 Table Univariate and multivariate regression analysis for log MAGE in non-DM low-BNP subgroup. (XLSX)

S2 Table Univariate and multivariate regression analysis for log MAGE in Non-DM high-BNP subgroup. (XLSX)

S3 Table Univariate and multivariate regression analysis for log MAGE in DM low-BNP subgroup. (XLSX)

S4 Table Univariate and multivariate regression analysis in DM high-BNP subgroup. (XLSX)
