# Supplementary figures and images for: Distinct impacts of sleep-disordered breathing on glycemic variability in patients with and without diabetes mellitus
Source: PLoS One. 2017 Dec 19;12(12):e0188689. doi: 10.1371/journal.pone.0188689 (PMC5736206; doi:10.1371/journal.pone.0188689)

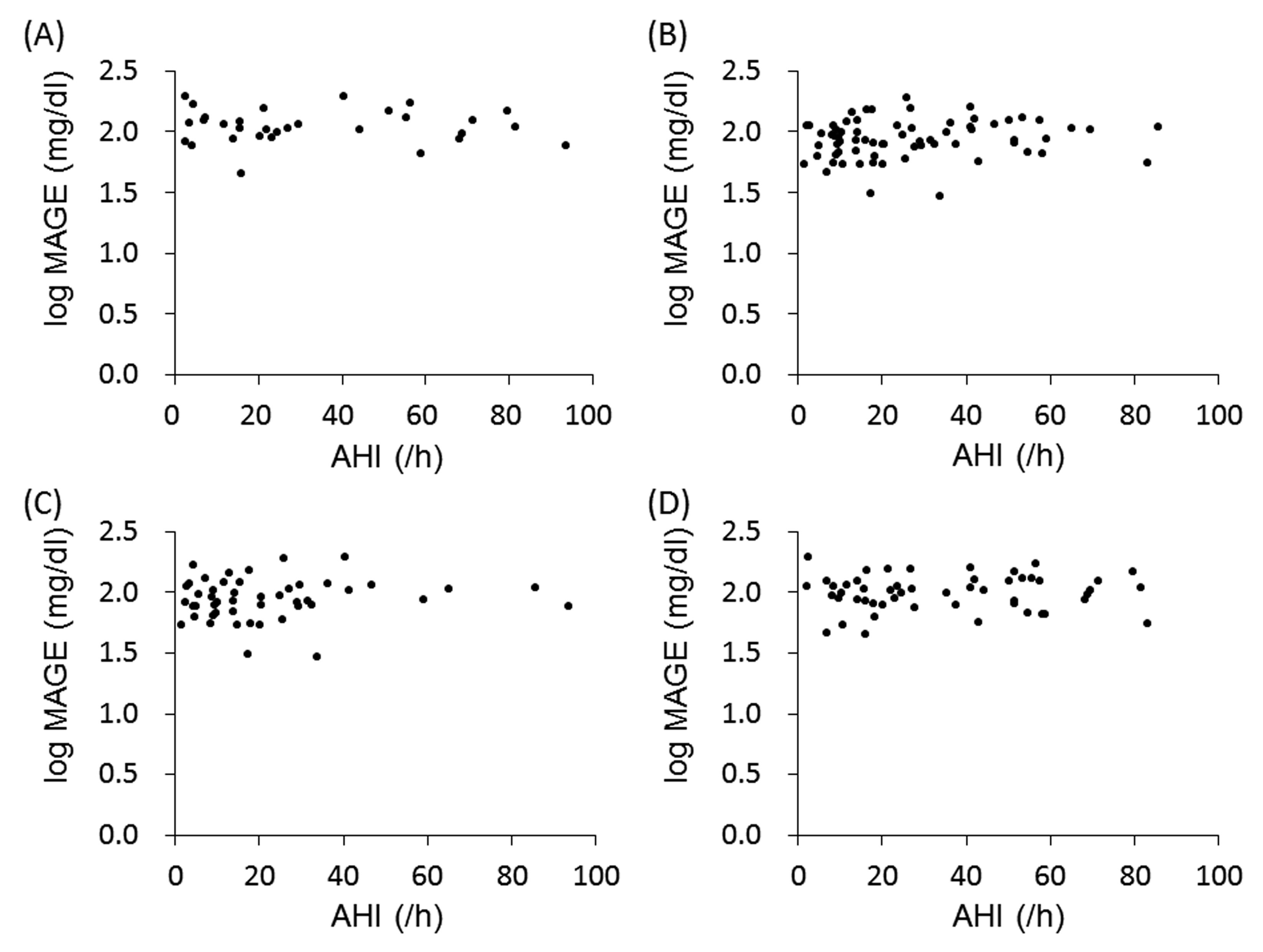

Supplement: S1 Fig — Scatterplots of log MAGE against AHI in the DM patients treated with insulin (A) and those without insulin (B). There was no correlation between AHI and log MAGE in the DM group regardless of insulin usage. Scatterplots of log MAGE against AHI in patients with short-duration DM (<7 years [median]: C) and those with long-duration DM (> 7 years [median]: D). Log MAGE was not correlated with AHI even in patients with short-duration DM. (TIF) [file pone.0188689.s001.tif]
